# A rare symptomatic giant multicystic dysplastic kidney in a 9-year-old boy: a case report and literature review

**DOI:** 10.1097/MS9.0000000000005468

**Published:** 2026-09-28

**Authors:** Hanan Joelle Ghanem, Mohammad Adi, Tareq Ibrahim Wehba, Yazan Soliman Khadour, Omar Mohammad Moussab Abo Jaish, Saleh Hamdi Hamdi, Fawaz Al Sharif

**Affiliations:** a Faculty of Medicine, Al Andalus University for Medical Sciences, Tartous, Syrian Arab Republic; b Faculty of Medicine, Damascus University, Damascus, Syrian Arab Republic; c Faculty of Medicine, Tishreen University, Lattakia, Syrian Arab Republic; d Faculty of Medicine, Tartous University, Tartous, Syrian Arab Republic; e Department of Internal Medicine, Damascus University, Damascus, Syrian Arab Republic; f Department of Urology, Damascus University, Damascus, Syrian Arab Republic; g Department of Pediatric Surgery, Damascus University, Damascus, Syrian Arab Republic

**Keywords:** case report, multicystic dysplastic kidney, nephrectomy, symptomatic MCDK

## Abstract

**Background:**

Multicystic dysplastic kidney (MCDK) is a congenital condition that presents as a dysmorphic kidney containing several non-functional cysts. It is typically asymptomatic but can cause varied manifestations determined by the size and location of the cysts, inducing complications due to compression of nearby organs.

**Materials and methods:**

We present a case of a previously healthy 9-year-old male who was admitted with respiratory and digestive manifestations. Imaging identified a large mass arising from a right MCDK with no functional renal tissue, causing compression of nearby organs. The symptoms due to mass effect necessitated complete surgical removal of the dysplastic kidney.

**Results:**

The surgery was successful. The child improved significantly postoperatively with the resolution of symptoms and restoration of organ function.

**Conclusion:**

This case highlights the importance of early diagnosis and surgical removal in cases that are symptomatic of MCDK. Some cases may manifest severe symptomatic consequences due to organ compression, emphasizing the significance of considering this strategy in cases with large MCDK.

## Introduction

Multicystic dysplastic kidney (MCDK) is a congenital disease in which the kidney contains multiple non-communicating cysts, with minimal to no functional renal tissue[1]. A study of 47 children reported male predominance (57.4%) and a higher incidence on the right side (55%)[2]. Bilateral MCDK occurs in about 20% of prenatally detected cases and is often incompatible with life[3]. MCDK is isolated in 70–75% of cases[4]. About 25% of cases show extrarenal abnormalities, which may involve the heart, brain, and digestive system. Patients may present with a concomitant urinary tract infection and/or hypertension. The cause of MCDK is still uncertain; the obstruction theory suggests that severe fetal obstructive hydronephrosis caused by renal atresia can lead to MCDK. The alternative theory postulates a disrupted interaction between the ureteric bud and renal precursor cells^5^. Most infants are asymptomatic. However, some may present with a painful flank mass, occasional hypertension, or urinary tract infection. The contralateral kidney may exhibit anomalies, such as vesicoureteral reflux (VUR), increasing the risk of developing pyelonephritis^[^1,5,6^]^. Renal ultrasound (US) is the gold standard for prenatal detection. In the postnatal phase, US, micturating cystourethrogram (MCUG), and nuclear imaging are useful for diagnosis^[^1,4,5^]^. Differential diagnosis includes autosomal recessive/dominant polycystic kidney disease, glomerular disease, obstructive dysplasia, medullary cystic dysplasia, hydronephrosis, polycystic nephroma, Wilms tumor, renal cell carcinoma, and clear cell sarcoma[3]. Conservative therapy is recommended in asymptomatic cases when neoplasia is excluded. A nephrectomy is necessary in symptomatic cases or when malignancy is suspected. Less invasive treatment options include sclerotherapy, which involves introducing a sclerosing material that deactivates the cyst’s secretory cells to prevent fluid reaccumulation[7]. Herein, we report a case of a child with a giant symptomatic MCDK that was treated surgically. This case report has been written in accordance with the SCARE 2025 criteria^8^.

HIGHLIGHTS

- A giant multicystic dysplastic kidney (MCDK) can present in late childhood with compressive symptoms.
- Surgical nephrectomy effectively resolves respiratory and gastrointestinal complications.
- Early detection and careful follow-up of MCDK are essential to preserve contralateral kidney function.

## Case presentation

A 9-year-old male, weighing 20 kg and previously asymptomatic with no recorded medical or family history, presented with increasing abdominal distention over the past 2 weeks, resulting in vomiting and increasing respiratory difficulty.

Physical examination revealed a distended abdomen and a right-sided abdominal mass, in addition to numerous cervical nodular enlargements indicative of inflammatory lymphadenopathy. On examination, the patient exhibited signs of respiratory distress, including tachypnea and the use of accessory muscles. The chest examination showed dulness to percussion in the right lower lung field and decreased breath sounds bilaterally. Laboratory parameters were within normal limits for creatinine at 0.45 mg/dL (normal: 0.3–0.7 mg/dL), with a mildly elevated erythrocyte sedimentation rate (ESR) of 35 mm/h (normal: 0–20 mm/h) and an alanine aminotransferase (ALT) level of 50 U/L (normal: 10–40 U/L).

Urinalysis showed clear urine with a pH of 6.0, a specific gravity of 1.020, and no leukocytes, nitrites, or protein. Microscopic examination of the urine was unremarkable.

Abdominal ultrasound (US) revealed a cystic structure occupying a substantial portion of the right abdomen and pelvis, measuring more than 25 cm in length, 17 cm in anteroposterior diameter, and 13 cm in width. A computed tomography (CT) scan showed a large cystic structure within the renal capsule that extended from the diaphragm to the pelvis, devoid of any recognizable functional renal tissue. This cystic mass compressed bowel loops, shifting them toward the left and pushing the liver upward (Fig. 1). Based on the imaging findings, a diagnosis of MCDK was confirmed. The contralateral kidney was normal in size, without evidence of hypertrophy, structural anomalies, or changes. DMSA renal scintigraphy and voiding cystourethrography could not be performed due to the unavailability of these diagnostic modalities. Consequently, the diagnosis and management were based on clinical evaluation and imaging findings.

**Figure 1.**
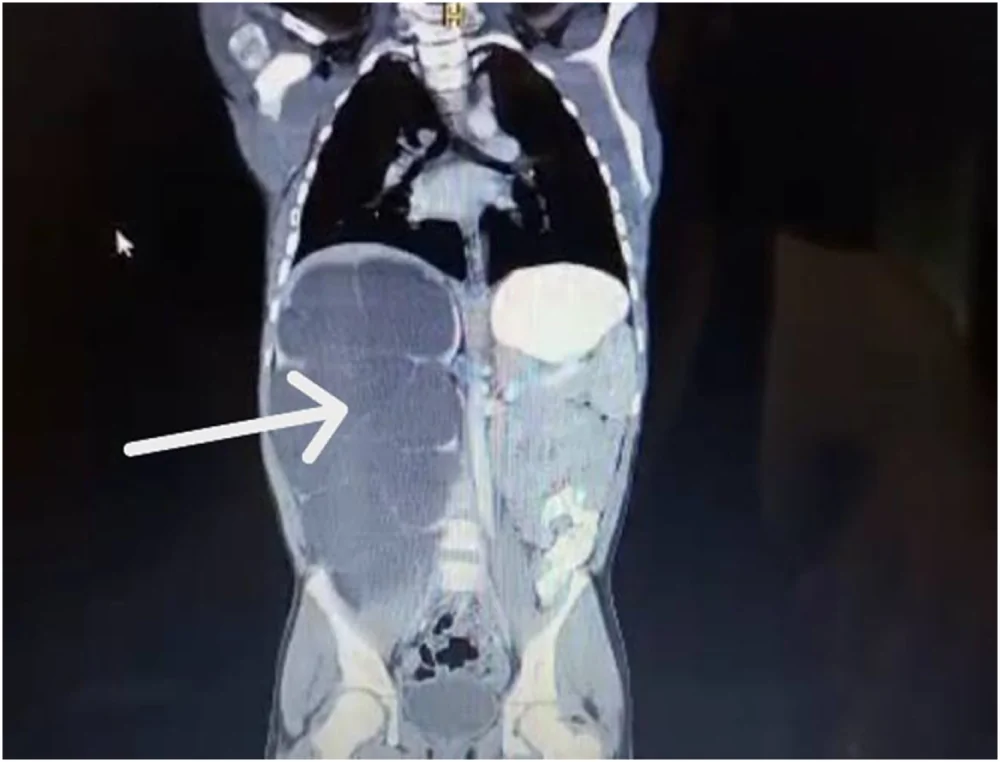
Coronal contrast-enhanced CT demonstrating a giant multicystic dysplastic right kidney (arrow) occupying most of the abdomen, displacing the liver superiorly and bowel loops medially.

The patient underwent a right nephrectomy through a subcostal incision.

The nephrectomy was performed by a pediatric surgery consultant at a tertiary referral center experienced in pediatric urologic procedures. A senior trainee assisted under supervision, and the case was conducted in collaboration with the pediatric urology team. Multiple non-communicating cysts of varying sizes were identified, consistent with a MCDK (Fig. 2).

**Figure 2.**
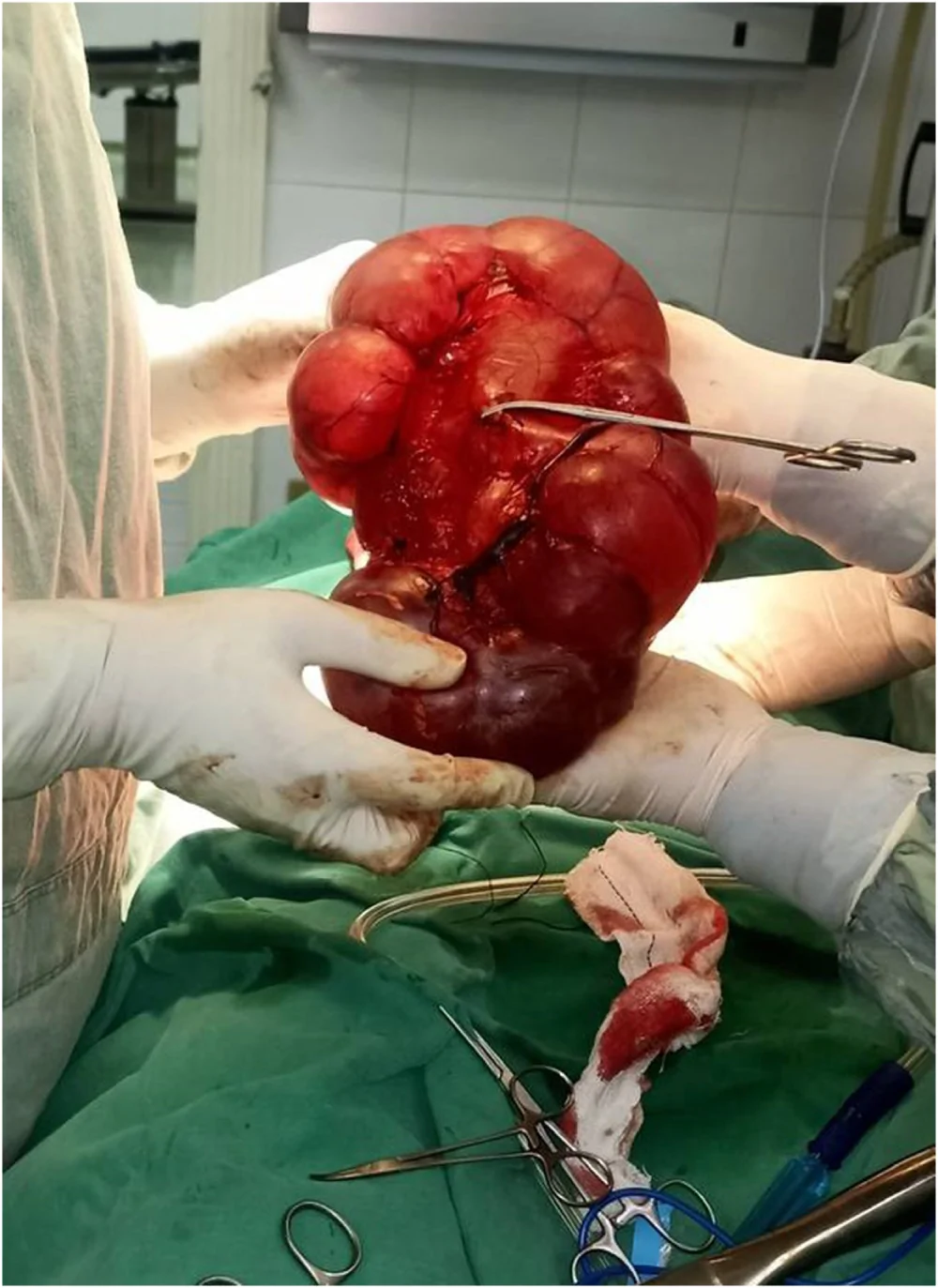
Intraoperative view of the resected multicystic dysplastic right kidney, showing multiple cystic cavities and a lobulated external surface.

The pathological examination revealed collapsed glomerular tufts, thickened Bowman’s capsules, and fibrosis in some glomeruli, along with chronic inflammation and fibrosis in the interstitium. These findings suggest chronic non-specific pyelonephritis. It also revealed a severely atrophic kidney with marked thinning of the parenchyma and chronic non-specific inflammatory cells. Postoperatively, the ESR and ALT returned to normal values, the patient’s respiratory and digestive symptoms resolved, and the child was discharged in good condition. Follow-up included clinical evaluation, blood pressure measurement, and renal ultrasound at 6 months. Long-term surveillance will follow the schedule proposed by Jawa *et al* (2021): ultrasounds at 6 months, 2, 5, 10, and 15 years.

## Discussion

MCDK is the second most common cause of abdominal mass in neonates[9]. It was initially described by Harley in 1864, and later by Schwartz in 1936[10]. As noted by Verghese *et al*, malformations in the contralateral kidney associated with MCDK may arise and require regular evaluation and management. VUR, which has been documented in 4% to 43% of MCDK cases, is the most clinically significant malformation in the contralateral kidney. Other anomalies, including ureteropelvic and ureterovesical junction obstructions, have been reported in 5% to 15% and 6% of cases, respectively[1]. In our case, the patient had MCDK without any concomitant abnormalities of the contralateral kidney or the urinary tract. MCDK typically manifests as a mass during infancy but may not be symptomatic until adulthood. However, in a few cases, the mass is large enough to cause respiratory, gastrointestinal, or other symptoms that could be life-threatening^[^11,12^]^. In our case, the patient was asymptomatic until the age of nine, when he complained of a distended abdomen with severe digestive and respiratory symptoms. Ultrasound imaging is the first-line approach for evaluating MCDK. It reveals a pattern of multiple non-communicating cysts of varying sizes, with absent kidney tissue. The widespread use of detailed prenatal ultrasounds around the 20th week of gestation has increased in-utero diagnoses[13], which has reduced the incidence of clinical symptoms such as abdominal masses and UTIs. Prenatal ultrasound identifies most MCDK cases, while postnatal detection accounts for 20% of cases. It is recommended that newborns suspected of having MCDK receive a confirmatory postnatal renal ultrasound before hospital discharge^[^1,11,14,15^]^. In our case, the diagnosis was established at the age of nine due to the symptoms. While conservative management is the primary approach for MCDK, surgery may be important in cases of abdominal or flank pain, recurrent UTIs, hypertension, suspected malignancy, and progressive renal enlargement resulting in respiratory or intestinal dysfunction^[^3,14^]^. In our case, surgical intervention was necessary due to the severity of the digestive and respiratory symptoms. During the procedure, the entire kidney and the ureter were removed after being isolated from neighboring structures. Following surgery, the symptoms disappeared. Pettit and Chalmers performed a systematic review of neonatal MCDK with mass effect. They found that aspiration or sclerotherapy often led to recurrence, confirming surgical nephrectomy as the definitive treatment[7]. Patients with compensatory hyperplasia or other hypoplastic kidney abnormalities should undergo close monitoring, including prompt evaluation for chronic kidney disease with serum creatinine. Due to the possibility of hyperfiltration and heightened renin activity in MCDK, there is a slightly elevated risk of hypertension later in life. Although this risk is low, it is advisable to conduct regular blood pressure checks[1]. In our case, we advised conducting an annual blood pressure evaluation and a renal ultrasound for the remaining kidney. According to Jawa *et al*, children with unilateral MCDK should undergo serial renal ultrasounds at 6 months, 2, 5, 10, and 15 years[16]. Although the solitary functioning kidney may initially appear normal, there is a higher risk for scarring, hypertrophy, or other late-onset abnormalities. For this reason, close monitoring is necessary. Relying solely on creatinine levels may not provide a comprehensive view of kidney health in individuals with MCDK. While creatinine indicates renal function, it does not reveal early structural changes. Therefore, ultrasound imaging is crucial, as it can detect anatomical alterations, offering a more thorough evaluation by monitoring both the function and the structure of the kidneys. Early identification of these anomalies using renal ultrasound can help preserve kidney function. Frequent monitoring ensures that any urgent problems with the remaining kidney are addressed in time. A comparison of similar published symptomatic or giant MCDK cases is shown in Table 1.

**Table 1:** Review of some similar cases.

† PN	Age/ gender	Age at diagnosis	Presentation	Exam description	Diagnostic tests	MCDK^††^ side	Management	MCDK^††^ size & other notes
[5]	9 years/ Female	Prenatal.	Asymptomatic until age 9, prompting a repeat ultrasound due to MCDK^††^ history.	NR‡	**USG^§^** 1 week postnatally: A 4.9-cm cystic structure in the left kidney. MAG-3 scans at 1 month: No functioning renal tissue in the left renal fossa. Repeat **USG§** at age 9: An 18-cm complex multicystic mass in the left kidney. **MRI**: Confirmed the USG§ findings.	Left	Laparoscopic left nephrectomy; *En bloc* removal via Pfannenstiel incision.	11 × 11 × 3.5 cm (191 g).
								Microscopic examination: Stratified squamous tissue with a keratin pearl.
								Final pathology: Dermoid cysts arising in an MCDK^††^.
[12]	Full-term newborn male	At birth	Respiratory distress	Distended abdomen with palpable abdominal; subcostal retractions	**X-ray** and **CT**^‡‡‡^ scan revealed a large multicystic kidney lesion (14x11.5x8.5 mm) arising from the right renal fossa, crossing the midline, and displacing bowel loops; explorative laparotomy confirmed the diagnosis of right	Right	O_2_ nasal prongs & nasogastric tube. Laparotomy; nephrectomy.	Largest cyst measured 16 cm × 16cm
[13]	9 months/ Female	Initial detection incidentally; confirmed at age 2	Left-sided abdominal lump; hypertensive	NR‡	**USG^§^**: Multicystic left renal mass.	Left	Amlodipine; chemotherapy; left open radical nephrectomy.	Initially 8.5 × 7.5 × 7.0 cm (BOSNIAK GRADE 3), later 14.8 × 12.3 × 11.3 cm.
					**CECT**^§§§^: at 9 M, multiseptated cystic mass; at 2 Y, well-defined multiloculated cystic lesion replacing the left kidney with mild enhancement of the walls and foci of calcification.			
								Histopathology confirmed MCDK^††^; asymptomatic and normotensive at 1 year of follow-up.
					**USG^§^ guided core cut biopsy**: Inconclusive			
[14]	5 Years/ Male		Recurrent UTI^§§^; history of rectourethral fistula; feces-stained urine; suspicion of right renal agenesis; duplex collecting system in the left kidney.	NR‡	**Urethrocystography**: A dilated posterior wall of the prostatic urethra; urethral diverticulum.	Right	Partial nephrectomy of the left kidney to remove the upper pole ureter.	0.91 × 1.21 × 1.91 cm
								No further follow-up imaging was provided; parents reported no complaints after surgery during follow-up.
					**MRI**: Retroperitoneal cluster of cystic structures in the right flank area; non-functional MCDK^††^; dilated, orthotopic ureter with a blind ending; left kidney with 2 ureters on the upper and middle pole.			
[15]	12 months/ NR‡	Prenatal with postnatal Confirmation.	Left-sided kidney dilation on prenatal ultrasound.	NR‡	**Postnatal USG**^§^: 1.5 cm heterogeneous focal area at the UPLK with cystic areas and discrete proximal ureter ectasia. **MRI:**	Left	Ultrasound follow-up.	Multicystic area with a global diameter of 2 cm (larger cyst: 9.5 mm).
								No duplicated collecting system was observed; a differential diagnosis with Wilms tumor was considered but not confirmed.
					Multiple cystic images at the UPLK.			
					**NSE**^‡‡^ **level** within normal limits. **Renal scintigraphy** was normal.			
	13 months/ NR‡	Prenatal ultrasound at 19 weeks of gestation.	A febrile urinary tract infection at 1 month of age.	NR‡	**USG§**: ectasia and hypoplasia of the upper moiety of the right kidney.	Right	Antibiotic prophylaxis with amoxicillin-clavulanic acid; ultrasound follow-up.	NR‡
					**MRI**: segmental MCDK^††^ affecting the upper moiety of a duplex right kidney and a 10 mm right ureterocele.			
					**ceVUS**†††: no vesicoureteral reflux.			
					**Scintigraphy**: smaller right kidney with 33.7% less function.			
					**USG^§^** at 13 months, no cystic image indicating dilated upper moiety was seen.			
^[^17^]^	7 months/ Female	Prenatal (16 weeks of gestation)	Asymptomatic; no developmental or psychomotor delays.	No palpable mass on examination	Multiple **USG**s^§^, **MRI**, and **laboratory tests** identified a cystic structure in the left adnexa measuring 1.5 × 0.8 × 1.3 cm.	Left	Surgical exploration at 7 months; removal of the cyst via trans-abdominal Pfannenstiel incision.	1.5 × 0.8 × 1.3 cm.
								Surgical pathology confirmed benign cystic tissue consistent with a cystic dysplastic kidney; postoperative follow-up is planned with a yearly renal ultrasound.
^[^18^]^	13 years/Female	Prenatal, around 26 weeks of gestation.	Increasing blood pressure around 1 month after birth gradually develops hypertension.	Normal development, normal weight and height, no surface malformation.	**Prenatal USG**^§^: abdominal mass lesions indicative of MCDK^††^.	Left	Benazepril hydrochloride; left nephrectomy at 7 months.	**Postoperative outcome**: Blood pressure normalized. Renin and aldosterone levels gradually reduced to normal. Serum creatinine level and eGFR within normal range.
					**Blood tests**: Elevated plasma renin activity and serum aldosterone levels.			
					**Abdominal USG^§^ & MRI**: atrophic left kidney with multiple cysts.			
					**Renal DTPA scintigraphy**: differential GFR indicating nonfunctional left kidney.			
								**Current Status**: Routine check-ups with blood pressure monitoring; not on any medications.

†PN, paper number; ‡NR, not reported; §USG, ultrasonography; MRI, magnetic resonance imaging; †† MCDK, multicystic dysplastic kidney; ‡‡NSE, neuron-specific enolase, §§UTI, urinary tract infection; UPLK, upper left kidney pole; †††ceVUS, contrast-enhanced voiding ultrasonography; ‡‡‡CT, computed tomography; §§§CECT, contrast enhanced computed tomography.

## Conclusion

We report a case of a giant, symptomatic MCDK in a 9-year-old boy, presenting with respiratory and digestive symptoms from mass effect, that was successfully managed with nephrectomy, resulting in complete symptom resolution and preservation of contralateral renal function. This outcome supports early diagnosis and prompt surgical management of symptomatic MCDK and highlights the value of prenatal detection in preventing delayed, complex presentations.

## Data Availability

All data relevant to this case report are included within the article. No additional datasets were generated or analyzed. Further de-identified data are available from the corresponding author upon reasonable request.

## Research registration unique identifying number (UIN)

Not applicable (this is a case report and does not require registration).

## Patient perspective

The patient’s guardian expressed satisfaction with the surgical outcome and agreed to share the case for educational purposes.
